# Assembly of Mitochondrial Genome of Oriental Plover (*Anarhynchus veredus*) and Phylogenetic Relationships Within the Charadriidae

**DOI:** 10.3390/genes16091030

**Published:** 2025-08-29

**Authors:** Baodong Yuan, Xuan Shao, Lingyi Wang, Jie Yang, Xiaolin Song, Huaming Zhong

**Affiliations:** 1 College of Agriculture and Biology, Liaocheng University, Liaocheng 252000, China; yuanbao365@163.com; 2 College of Biology and Food, Shangqiu Normal University, Shangqiu 476000, China; 18769787092@163.com (X.S.); 13333982035@163.com (L.W.); yangjie201604@163.com (J.Y.); yaoyaolin1993@163.com (X.S.)

**Keywords:** *Anarhynchus veredus*, Charadriiformes, Charadriidae, mitochondrial genome, phylogeny

## Abstract

**Background:** Traditional morphology-based classification of the Oriental Plover (*Anarhynchus veredus*) is inconsistent with molecular evidence, underscoring the necessity of incorporating molecular data to elucidate its evolutionary relationships within Charadriidae. **Methods:** Here, we present the first complete mitochondrial genome of *A. veredus* by Illumina NovaSeq Sequencing and explore its evolutionary implications within Charadriidae. **Results:** The mitogenome spans 16,886 bp and exhibits conserved structural features typical of Charadriidae, including gene order, overlapping coding regions, and intergenic spacers. Nucleotide composition analysis revealed a GC content of 44.3%, aligning with other Charadriidae species (44.5–45.8%), and hierarchical GC distribution across *rRNA*, *tRNA*, and protein-coding genes (PCGs) reflects structural and functional optimization. Evolutionary rate heterogeneity was observed among PCGs, with *ATP8* and *ND6* showing accelerated substitution rates (Ka/Ks = 0.1748 and 0.1352) and *COX2* under strong purifying selection (Ka/Ks = 0.0678). Notably, a conserved translational frameshift in *ND3* (position 174) was identified. Phylogenetic analyses (ML/NJ) of 88 Charadriiformes species recovered robust topologies, confirming that the division of Charadriidae into four monophyletic clades (*Pluvialis*, *Vanellus*, *Charadrius*, and *Anarhynchus*) and supporting the reclassification of *A. veredus* under *Anarhynchus*. **Conclusions:** This study resolves the systematic position of *A. veredus* and highlights the interplay between conserved mitochondrial architecture and lineage-specific adaptations in shaping shorebird evolution.

## 1. Introduction

Shorebirds (Charadriiformes) are an ecologically diverse and globally distributed order of birds, comprising approximately 390 species, and represent one of the most ecologically and morphologically diverse avian radiations [1,2]. The family Charadriidae, comprising plovers and lapwings, represents a globally distributed group of shorebirds occupying diverse ecosystems from coastal wetlands to arid grasslands. These medium-sized waders exhibit remarkable ecological adaptability, as evidenced by their radiation across habitats spanning Arctic tundra to tropical mudflats [3]. Among them, the Oriental Plover (*Anarhynchus veredus*) stands out as a long-distance migrant that breeds in Mongolian grasslands and winters in northern Australia. This species demonstrates unique ecological specialization through its sexually dimorphic breeding plumage and adaptations particularly suited to sparse vegetation zones where cryptic coloration enhances predator avoidance [4]. Although currently classified as Least Concern by the IUCN, its dependence on ephemeral breeding habitats renders it vulnerable to climate-driven aridification and anthropogenic landscape modifications.

Recent molecular systematic studies have substantially revised our understanding of Charadriidae phylogeny over the past two decades, particularly regarding genus-level classifications and polyphyly resolution [5,6,7,8,9]. Dos Remedios et al. (2015) sampled two mitochondrial genes (*COI* and *ND3*) and four nuclear loci (*ADH5*, *FIB7*, *MYO2*, and *RAG1*) from 29 *Charadrius* species [7]. Their MCC tree divided the genus *Charadrius* into two major clades (*Charadrius* clade I and II, i.e., CRD I and CRD II), which were further categorized into six minor clades (Clades a–f) of sister species. Among these, the Oriental Plover was clustered into the CRD II-b clade. Phylogeny result from eight mitochondrial and two nuclear loci further support that the Oriental Plover belongs to CRD II [10]. Černý & Natale utilized 27 genetic loci and a matrix of 69 skeletal characters to address the relationships of most Charadriiformes species [11]. Sun et al. (2024) constructed a phylogenetic tree based on complete mitogenomes using sequences of 13 mitochondrial protein-coding genes (CDSs) from 114 available Charadriiformes mitogenomes [12]. Although both studies strongly support the recognition of three major shorebird clades and the division of Charadriidae into monophyletic genera (including *Anarhynchus*, *Vanellus*, *Charadrius*, and *Pluvialis*), the Oriental Plover was not included in either analysis.

Mitochondrial genomes offer unique advantages for resolving such taxonomic uncertainties. Their maternal inheritance circumvents complexities of biparental recombination, while differential evolutionary rates across genomic regions enable phylogenetic resolution at multiple timescales: rapidly evolving control regions elucidate recent divergences, whereas conserved protein-coding genes like *COX1* retain deep phylogenetic signals [13,14]. The compact size (16–18 kb) of animal mitogenomes, combined with heterogeneous evolutionary rates among their 37 genes, provides multiple markers for robust branch support estimation [15,16]. However, the lack of a complete mitogenome for *A. veredus* highlights the need for these data to resolve its phylogenetic relationships within the Charadriidae.

In this study, we assembled and annotated the first complete mitochondrial genome of *A. veredus* using Illumina NovaSeq technology. To investigate the interspecific variation in Charadriidae, we compared mitogenomes from 13 Charadriidae mitogenomes to identify lineage-specific adaptations in base composition, codon usage, gene arrangement, and selection pressures. Furthermore, we reconstructed a mitogenome-based phylogeny to clarify the evolutionary position of *A. veredus* within Charadriidae.

## 2. Materials and Methods

### 2.1. Specimen Collection and DNA Extraction

One *A. veredus* individual was collected on 8 July 2024, in Weihai City, Shandong Province, China (37.19° N, 122.23° E). The species identification was made based on morphological characteristics, following a Field Guide to the Birds of China [17]. Leg muscle was sampled and preserved in 95% ethanol. All experimental procedures were conducted in accordance with the guidelines established by the Ethics Committee of the Animal Experiments of Shangqiu Normal University (Shangqiu City, Henan, China) and were approved under protocol number 2024016. Total genomic DNA was extracted from muscle tissue samples using the E.Z.N.A.^®^ Tissue DNA Kit (Omega, Norcross, GA, USA), following the manufacturer’s protocol.

### 2.2. Library Construction and Illumina NovaSeq Sequencing

DNA integrity was verified by 1% agarose gel electrophoresis, and quantification was performed using a NanoDrop 2000 spectrophotometer (Thermo Fisher Scientific, Waltham, MA, USA). Sequencing libraries were prepared from 1 μg of high-quality genomic DNA using the TruSeq™ Nano DNA Sample Prep Kit (Illumina, San Diego, CA, USA). Genomic DNA was fragmented to a target size of 300–500 bp using the Covaris M220 ultrasonicator (Covaris, Shelton, CT, USA). The fragmented DNA underwent three consecutive enzymatic reactions: (1) end repair to generate blunt-ended fragments, (2) 3′ adenylation to facilitate adapter ligation, and (3) ligation of uniquely indexed Illumina adapters using the TruSeq™ Nano DNA Sample Prep Kit. The adapter-ligated DNA was amplified through 8 cycles of PCR amplification. PCR products were size-selected (300–500 bp) by electrophoresis on 2% Certified Low Range Ultra Agarose (Bio-Rad, Hercules, CA, USA) at 120 V for 40 min, followed by gel extraction using the QIAquick Gel Extraction Kit (Qiagen, Hilden, Germany). Library quantification was performed using the TBS380 PicoGreen dsDNA Assay Kit (Invitrogen, Carlsbad, CA, USA) with a Qubit 3.0 Fluorometer (Thermo Fisher Scientific, Waltham, MA, USA). Cluster generation was achieved through bridge amplification on an Illumina cBot system using the TruSeq PE Cluster Kit v3-cBot-HS (Illumina, San Diego, CA, USA). Paired-end sequencing (2 × 150 bp) was conducted on the Illumina NovaSeq 6000 platform (Illumina, San Diego, CA, USA) with the TruSeq SBS Kit v3 (300 cycles). Image analysis and base calling were performed using NovaSeq Control Software v1.7.0 (Illumina, San Diego, CA, USA).

### 2.3. Quality Control and Preprocessing of the Sequencing Data

Raw sequencing reads were processed through a multi-stage quality control pipeline using Trimmomatic v0.39 [18]. Adapter sequences were eliminated through automated alignment, and 5′-terminal nucleotides containing non-canonical bases (non-AGCT) were precisely excised. A sliding window algorithm (window size: 4 bp, step: 1 bp) subsequently removed terminal regions with averaged Phred scores <Q20, while reads containing >10% ambiguous nucleotides (N) underwent rigorous exclusion. Ultimately, processed reads shorter than 75 bp (<0.5× read length) were filtered through size-selection protocols.

### 2.4. Mitogenome Assembly and Annotation

The mitochondrial genome assembly of *A. veredus* was performed using GetOrganelle v1.7.5 software (https://github.com/Kinggerm/GetOrganelle, accessed on 12 September 2024) [19]. Candidate sequences were selected based on sufficient depth of coverage and longer assembly lengths, followed by alignment with the NCBI NT database to identify mitochondrial scaffolds. The selected sequences were then systematically assembled through precise overlapping regions. Subsequently, the MITOS software (http://mitos.bioinf.uni-leipzig.de/index.py, accessed on 15 September 2024) [20] was used to predict the protein-coding, *tRNA*, and *rRNA* genes of the mitochondrial genome, with the vertebrate mitochondrial code selected as the codon table. Finally, the CGView software [21] (http://stothard.afns.ualberta.ca/cgview_server/, accessed on 17 September 2024) was used to display the circular map of the genome.

### 2.5. Comparative Mitogenomic Analyses

The complete mitochondrial genomes of 13 Charadriidae species were selected for comparative analysis. The corresponding accession numbers are provided in Supplementary Material Table S1. The relative synonymous codon usage (RSCU) was calculated using PhyloSuite [22,23] and visualized with the ggplot2 package in R software (version 4.1.3). Nucleotide compositional biases were assessed using AT skew [(A − T)/(A + T)] and GC skew [(G − C)/(G + C)] [24]. To gain a deeper understanding of the selective pressure on protein-coding genes (PCGs), we calculated the evolutionary rates for each PCG within the Charadriidae family with PhyloSuite v1.2.3 [22,23]. Additionally, synteny analysis was conducted using Mauve software v2.4 [25], based on 13 Charadriidae mitochondrial genomes.

### 2.6. Phylogenetic Analyses

We first searched and retrieved available mitogenome sequences in GenBank format from the NCBI database. A total of 88 sequences from 88 Charadriiformes species, representing 44 genera across 12 families, along with the outgroup species *Balearica regulorum* (Gruiformes) were included in the phylogenetic analysis (Supplementary Material Table S1). PhyloSuite v1.2.3 [22,23] was used to extract the gene sequences from all 89 mitogenomes. The PCGs, *12S rRNA* and *16S rRNA* genes were aligned with MAFFT v7.505 [26]. For the PCGs, a codon-based alignment was performed using the ‘E-INS-i (accurate)’ strategy, while the rRNA genes were aligned under the same strategy but in normal alignment mode. Subsequently, the PCGs alignment results were refined using the codon-aware program MACSE v2.06 [27], which preserves reading frame and allows incorporation of sequencing errors or sequences with frameshifts. Finally, the aligned PCGs, *12S rRNA* and *16S rRNA* from the same species were concatenated together by PhyloSuite. ModelFinder v2.2.0 [28] was used to determine the best-fit partition model (Edge-unlinked) for phylogenetic analysis based on the Bayesian inference (BI) method, with the selection guided by the Bayesian Information Criterion (BIC). The model identified as the best fit for BI was GTR+F+I+G4. Bayesian inference phylogenies were inferred using MrBayes v3.2.7a [29] under a partition model (4 parallel runs, 28,000 generations), with the initial 5000 sampled data were discarded as burn-in. Additionally, neighbor-joining (NJ) phylogenetic analyses [30] were performed in MEGA X [31]. The percentage of replicate trees in which the associated taxa clustered together was determined using a bootstrap test with 10,000 replicates. Evolutionary distances were computed using the Jukes-Cantor method [32], expressed as the number of base substitutions per site. Rate variation among sites was modeled using a gamma distribution (shape parameter = 1). All ambiguous positions were removed for each sequence pair under the pairwise deletion option. The phylogenetic tree was visualized using FigTree v1.44 and further refined with Adobe Illustrator CS6 v16.0.0.

## 3. Results

### 3.1. Mitochondrial Genome Structure and Composition

**Figure 1:**
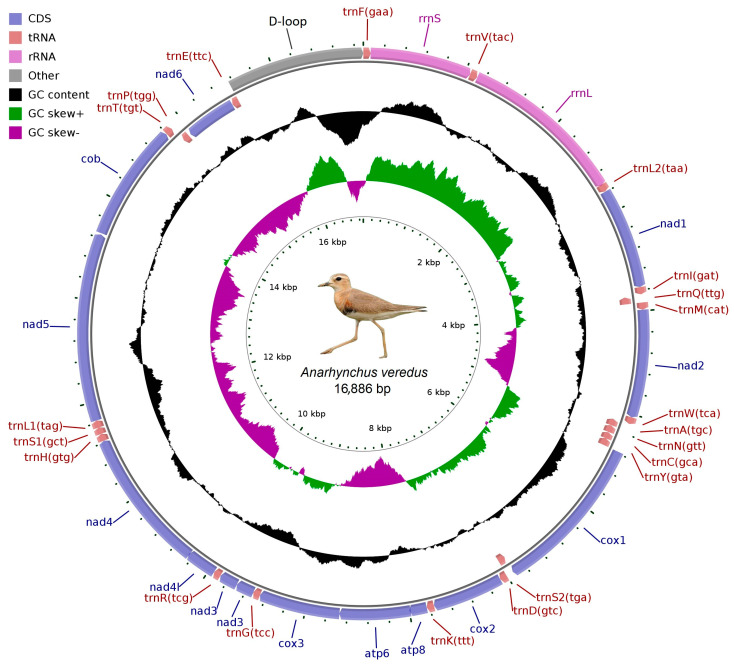
Circular map of the mitogenome of *A. veredus*. Genes in the outermost circle are transcribed in a clockwise direction, while those in the inner circle are transcribed counterclockwise. The inner circles display GC skew and G + C content.

We sequenced and assembled the mitogenome of *A. veredus* using Illumina NovaSeq technology, generating a total of 48,624,872 raw reads (Supplementary Material Table S2). The resulting sequence data were deposited in GenBank under the accession number SRR32628449. Following quality control, a total of 48,569,886 clean reads were retained, representing 7283.1 Mbp of clean data for further analysis (Supplementary Material Table S2). The *A. veredus* mitogenome was deposited in GenBank under accession numbers PV262314. Summary information is shown in Table 1. The *A. veredus* mitogenome possesses circular double-stranded DNA molecules, containing 13 PCGs, 22 *tRNA* genes, 2 *rRNA* genes. Among these, 9 genes (*tRNA^GLN^, tRNA^ALA^, tRNA^ASN^, tRNA^CYS^, tRNA^TYR^, tRNA^SER^, tRNA^PRO^, NAD6*, and *tRNA^GLU^*) were located on the light strand, while the remaining 28 genes were situated on the heavy strand. The D-loop region was located between *tRNA^GLU^* and *tRNA^PHE^* (Figure 1). In addition, we identified nine overlapping coding regions and 19 intergenic spacers. The largest overlapping coding region was 10 bp, located between *ATP8* and *ATP6*, while the largest intergenic spacer was 22 bp, positioned between *tRNA^PRO^* and *ND6*.

The GC nucleotide proportion in the complete mitochondrial genome of *A. veredus* was 44.3%, lower than the AT nucleotide proportion (Table 2). The AT skew for the complete mitochondrial genome was 0.131, while the GC skew was −0.389. In PCGs, tRNAs, rRNAs, and D-loop, the AT nucleotide proportion exceeded that of GC nucleotides. The AT skew was slightly positive, whereas the GC skew was negative (Table 2). These results indicate a higher abundance of A over T and C over G.

### 3.2. PCGs and Codon Usage

**Figure 2:**
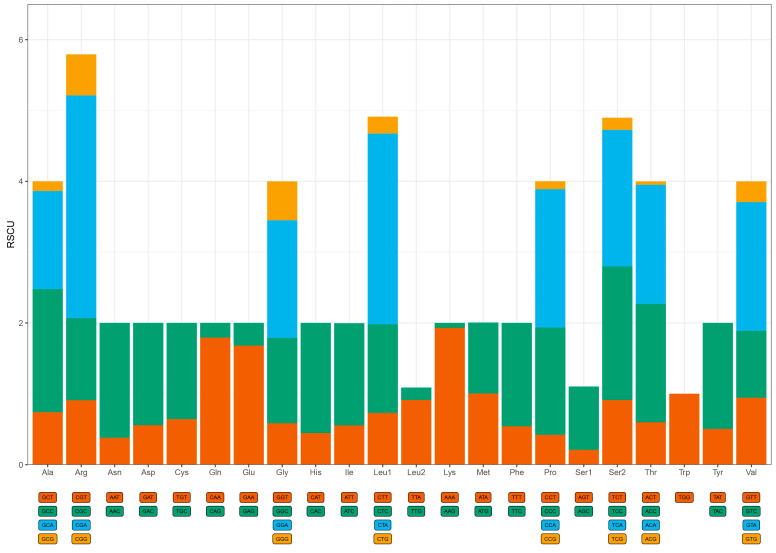
Relative synonymous codon usage (RSCU) of *A. veredus*. The *x*-axis indicates the amino acids encoded by the respective codons, with each amino acid labeled below. Corresponding RSCU values are plotted on the *y*-axis.

The initiation codon for most PCGs was ATG (N = 10), while the remaining three genes utilized alternative start codons: *COX1* and *ND5* used GTG, and *ND3* used ATC. The termination codons were TAA (N = 7), TAG (N = 2), T-- (N = 2), and AGG (N = 2). Additionally, an extra nucleotide (C: cytosine) was present at the position 174 of the *ND3* gene (Table 1). We conducted RSCU analysis to elucidate the codon usage bias of PCGs. The results revealed that Arg, Leu, Pro, Lys, and Ser were the most abundant amino acids (Figure 2). The five most prevalent codons were Arg (CGA), Leu (CUA), Pro (CCU), Lys (AAA), and Ser (UCA). The RSCU values indicated a tendency towards higher usage of A and C compared to T and G in most codons, while Gln, Cys, and Ser1 exhibited a preference for G in their codon usage.

### 3.3. Transfer and Ribosomal RNA Genes

**Figure 3:**
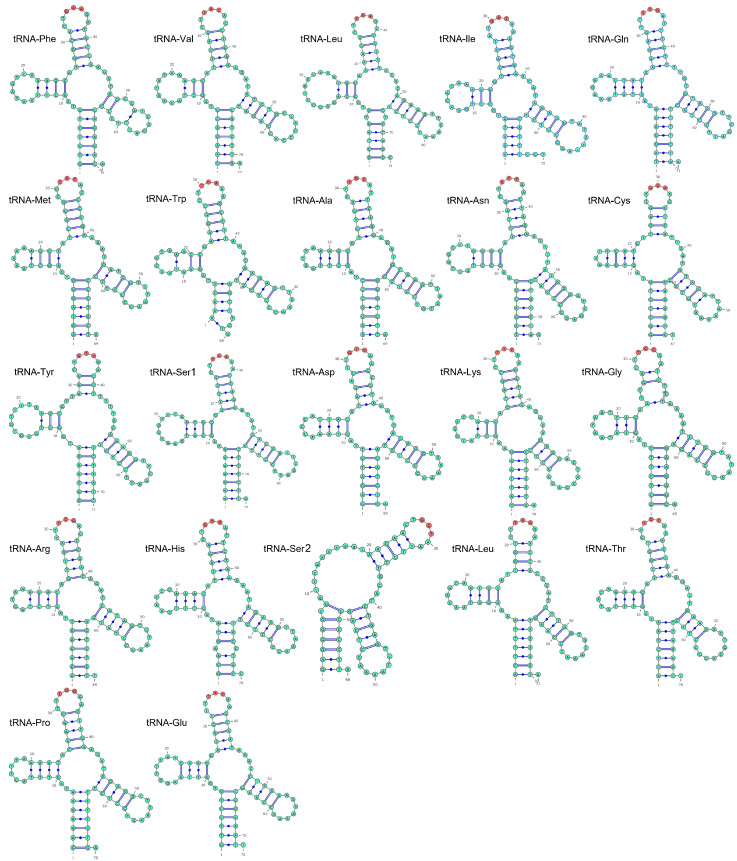
The predicted secondary structures of 22 tRNA genes in the *A. veredus* mitochondrial genome.

The mitogenome of *A. veredus* contained 22 transfer RNA genes, as in most vertebrates. The total lengths of *tRNAs* was 1546 bp, and these *tRNA* genes ranged from 66–74 bp. We found that only tRNA^SER2(GCT)^ lacked the dihydrouridine (DHU) arm, and the remaining 21 *tRNA* genes formed a typical cloverleaf structure (Figure 3). The *12S* and *16S rRNA* genes on the N-strand of *A. veredus* mitogenome were located between *tRNA^PHE^* and *tRNA^VAL^* and *tRNA^LEU^* (Table 1). Their lengths were 979 bp and 1601 bp, respectively. Both genes exhibited a negative AT skew and a positive GC skew (Table 2).

### 3.4. Control Region

The control region, also called the AT-rich region, is the longest non-coding region involved in mitogenomic replication and transcription. In the *A. veredus* mitochondrial genome, this region was located between the *tRNA^GLU^* and *tRNA^PHE^*. The length of the control region was 1321 bp, exhibiting a positive AT skew (0.051) and negative GC skew (−0.372) (Table 2).

### 3.5. Comparative Analysis of the Charadriidae Mitochondrial Genomes

The total length of 13 Charadriidae species mitogenomes varied from 15,723 bp (*Pluvialis apricaria*) to 17,074 bp (*Vanellus cinereus*). PCGs exhibited remarkable length conservation (11,385–11,397 bp; CV = 0.3%, Table 3). Structural RNAs exhibited moderate variation. *rRNA* lengths ranged from 2536 to 2583 bp (CV = 0.6%), and tRNA clusters varying between 1543 bp (*A. montanus*) and 1553 bp (*A. leschenaultia*). GC content stratification was evident across functional regions, with *rRNA* maintaining the highest GC levels (45.2–46.7%), followed by PCGs (44.6–46.8%) and tRNA (41.2–42.7%). Notably, species in the genus *Pluvialis* exhibited a higher genomic GC content (45.4–45.8%) than those in the genus *Vanellus* (44.6–44.9%). PCGs showed the greatest intergeneric divergence, with GC content ranging from 45.9% to 46.8% in *Pluvialis* and 44.8% to 45.3% in *Vanellus* (Table 3).

Strand asymmetry analysis revealed consistent positive AT-skew (0.131–0.153) and negative GC-skew (−0.402 to−0.374) across the entire genome (Table 3), which is in agreement with the characteristic light-strand compositional bias. Notably, *rRNA* genes displayed higher AT-skew (0.216–0.237) than PCGs (0.060–0.082). A notable exception was observed in *Charadrius dubius* and *Charadrius vociferus*, which exhibited positive tRNA GC-skew (0.017 and 0.005), contrasting with the consistently negative values found across other taxa. Additionally, a conserved frameshift at position 174 of the ND3 gene was observed in all 13 species.

**Figure 4:**
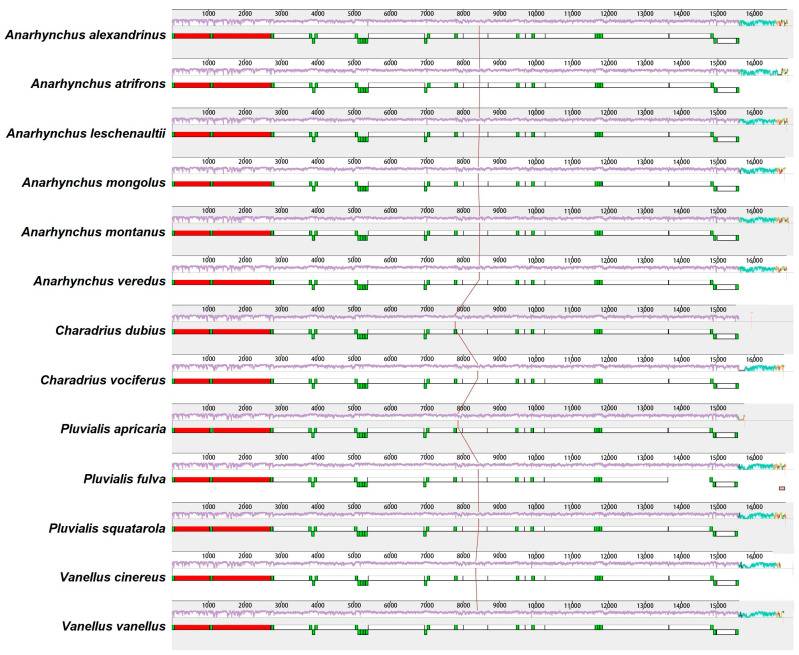
Gene arrangement comparison among the 13 Charadriidae species. The PCGs are represented in the white blocks, the *12S rRNA* and *16S rRNA* genes are indicated in the red blocks, and the *tRNA* genes are shown in the green blocks.

Comparative alignment of the 13 mitochondrial genomes showed that the gene order was conserved among *A. veredus* and their close Charadriidae relatives (Figure 4).

**Figure 5:**
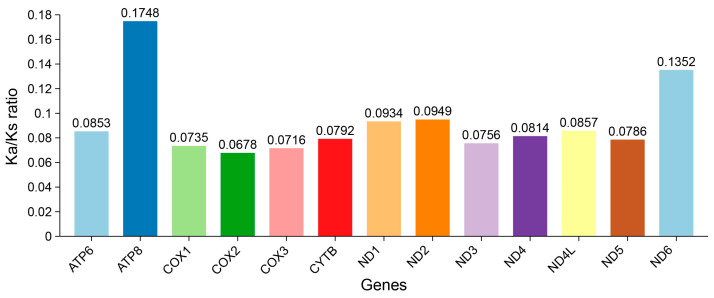
The ratio of non-synonymous substitutions to synonymous substitutions of PCGs based on 13 Charadriidae mitochondrial genomes.

To investigate the selective pressure acting up on PCGs within Charadriidae, the evolutionary rates of each PCG were calculated and compared. For all PCGs, the Ka/Ks ratios were consistently lower than 1 (Figure 5), indicating that purifying selection acted on all PCGs. *ATP8* and *ND6* exhibited high substitution rates (0.1748 and 0.1352, respectively), whereas *COX2* showed a low evolutionary rate (0.0678).

### 3.6. Phylogeny Analysis

**Figure 6:**
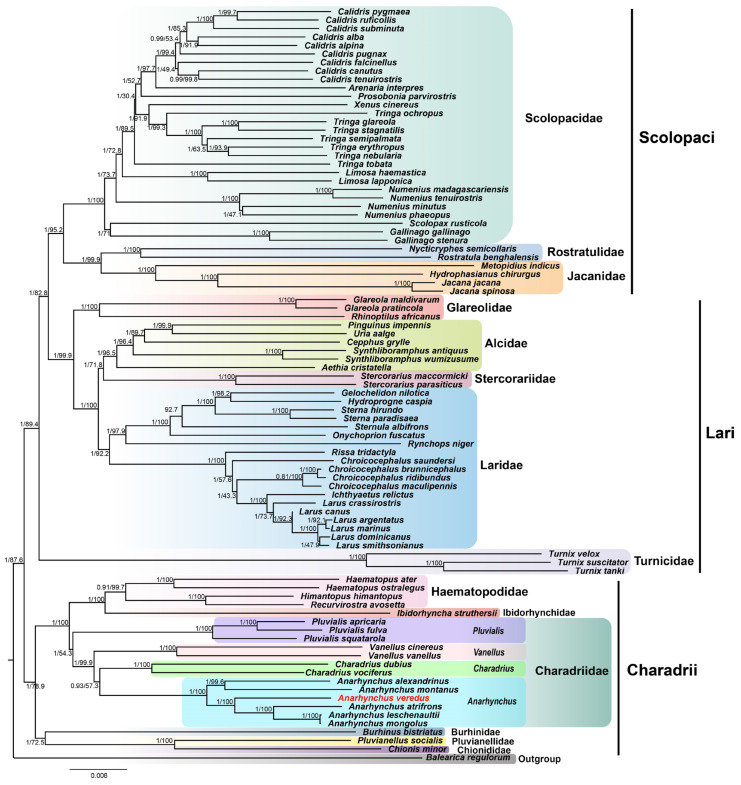
The NJ and BI phylogenetic tree of 88 Charadriiformes species based on 13 PCGs and 2 *rRNA* genes. *Balearica regulorum* in Gruiformes was designated as the outgroup species. The numbers above the branches represent posterior probabilities (BI) and bootstrap support values (NJ), respectively. The families and genera to which the species belong are displayed in colored background boxes. The oriental plover is marked in red.

Utilizing 13 PCGs, *12S rRNA*, and *16S rRNA* genes, we constructed phylogenetic trees for 88 Charadriiform species using both ML and NJ methods. The resulting topologies from both methods were found to be identical, with high support bootstrap values and posterior probability (Figure 6). This consistency of trees between ML and NJ approaches suggests a robust dataset and a reliable phylogenetic structure, reinforcing the credibility of the inferred evolutionary relationships. All the Charadriiformes groups form a strictly monophyletic group and were separated from the outgroup Gruiformes, verifying the reliability of Charadriiform as an independent evolutionary branch. The results showed that the Charadriiform were divided into three suborders: Scolopaci, Lari, and Charadrii. Lari and Scolopaci form a sister group, while Charadrii constitutes a separate branch. Among Scolopaci, family Jacanidae was closely related to Rostratulidae, whereas most species were classified within Scolopacidae Family. In the suborder Lari, Family Turnicidae was the earliest-diverging lineage and is positioned on a long evolutionary branch. Meanwhile, families Glareolidae, Alcidae, Stercorariidae, and Laridae formed a distinct clade in the phylogenetic tree. The 21 species of the Charadrii suborder were classified into six families: Haematopodidae, Ibidorhynchidae, Charadriidae, Burhinidae, Pluvianellidae, and Chionididae. Within this suborder, family Burhinidae, Pluvianellidae, and Chionidae formed a well-supported clade, which was the sister group to all other members of Charadrii. Notably, the ibisbill (*Ibidorhyncha struthersii*) and Magellanic Plover (*Pluvianellus socialis*) represent the sole species within the monotypic families Ibidorhynchidae and Pluvianellidae, respectively.

The family Charadriidae comprises four main genera: *Pluvialis*, *Vanellus*, *Charadrius*, and *Anarhynchus* (Figure 6). Among these, *Pluvialis* represents the basal lineage, having diverged first. Following this, *Vanellus* diverged, while *Charadrius* and *Anarhynchus* form a sister group. Our results support the monophyly of *Anarhynchus* and include the following species: Oriental Plover (*A. veredus*), Kentish Plover (*A. alexandrinus*), Black-fronted Dotterel (*A. atrifrons*), Greater Sand Plover (*A. leschenaultia*), Mountain Plover (*A. montanus*), and Mongolian Plover (*A. mongolus*).

## 4. Discussion

In this study, we report the complete mitochondrial genome of *A. veredus* for the first time. The total length of the *A. veredus* mitogenome was 16,886 bp, which falls within the range for other Charadriidae birds (15,723–17,074 bp). The gene arrangements of the 13 Charadriidae mitogenomes were highly conserved and largely identical to those of most avian species [33,34,35]. The *A. veredus* mitogenome displayed a typical, highly conserved architecture characteristic of Charadriidae. Gene order, overlapping regions (e.g., the 10 bp overlap between *ATP8* and *ATP6*), and intergenic spacers mirrored those of *A. alexandrines* [36] and *A. atrifrons* [37], suggesting minimal structural reorganization over evolutionary timescales.

Beyond structural conservation, nucleotide composition analysis revealed critical evolutionary patterns. The GC content of *A. veredus* mitogenome was 44.3%, aligning closely with other Charadriidae species (44.5–45.8%). These slight variations likely arose from differential mutation rates or selective constraints imposed by environmental factors, which may affect genome stability and function [38,39]. All studied Charadriidae species exhibited lower GC content than AT content, a pattern consistent with most avian mitogenomes [40,41]. The hierarchical GC distribution— characterized by high in *rRNA* and low in *tRNA*—likely reflects structural optimization. Elevated GC content in *rRNA* stabilizes ribosomal secondary structures through enhanced base-pairing interactions [42], while reduced GC content in *tRNA* may enhance D-loop flexibility during cloverleaf formation [43]. The significant GC divergence in PCGs between *Pluvialis* and *Vanellus* may reflect differential codon usage optimization, potentially associated with thermal adaptation or oxidative stress tolerance in their distinct ecological niches. The universal AT/GC skew polarity reflects conserved replication strand asymmetry [44]. However, the pronounced AT-skew in *rRNA* suggests transcription-driven mutational biases, as continuous transcription of rDNA could potentially increase deamination rates on the heavy strand [45]. Intergeneric comparisons indicated that *Anarhynchus* species exhibited higher PCGs GC-skew values (−0.399 to −0.409) than *Pluvialis* (−0.413 to −0.424) and *Vanellus* (−0.410 to −0.418), supporting their phylogenetic placement. These results underscore the balance in shorebird mitogenomic architecture between conserved functional elements and flexible non-coding regions, facilitating both metabolic stability and lineage-specific adaptations [11].

Functional constraints on mitochondrial genomes extended beyond nucleotide composition to protein-coding genes (PCGs). The PCGs of Charadriidae mitochondria exhibited high conservation, with their structure and arrangement aligning perfectly with prior studies on avian mitochondrial PCGs [33,34,35]. The conserved PCGs architecture highlights evolutionary constraints imposed by mitochondrial energy metabolism [46], while variable non-coding regions may mediate lineage-specific regulatory adaptations [13]. Among the 13 PCGs, canonical start codons (ATG) dominated (10/13), while *COX1* and *ND5* initiated with GTG and *ND3* with ATC—a pattern consistent across all 13 Charadriidae species except for *ND3* (ATT) in Killdeer (*C. vociferous*). These non-canonical codons may undergo post-transcriptional modification to ensure translational fidelity, a mechanism observed in avian mitochondrial systems [47]. Termination codons predominantly included TAA (7/13) and incomplete T-- (2/13), the latter resolved via mRNA polyadenylation, a ubiquitous feature of avian mitogenomes [24]. In metazoan mitochondrial genomes [48], incomplete stop codons (e.g., TA or T) are common and may be completed to TAA during mRNA maturation through post-transcriptional modification [49]. RSCU analysis revealed pronounced biases toward A/C-rich codons (e.g., *Arg-CGA*, *Leu1-CUA*), correlating with the overall A + T skew and mirroring trends in Little Ringed Plover (*C. dubius*) and Eurasian Oystercatcher (*Haematopus ostralegus*) [33]. Such codon preferences may reflect selection for translational efficiency or mutational biases during replication [50]. Evolutionary rate heterogeneity was evident: *ATP8* and *ND6* exhibited accelerated substitution rates (Ka/Ks = 0.1748 and 0.1352), while *COX2* showed the signature of strong purifying selection (Ka/Ks = 0.0678). This result aligns with findings in Kentish Plover (*A. alexandrinus*), where *ATP8*’s functional redundancy permits higher plasticity, whereas *COX2*’s role in electron transport imposes strict conservation [12,36].

In addition to sequence-level conservation, we identified a notable genomic feature in *A. veredus*. The nad3-174 frameshift observed in *A. veredus* has also been found in many Archosauria-Testudines species [47,51,52,53,54]. Computational analysis of the nucleotide sequence surrounding position 174 reveals a conserved motif (TTC-CTA-GTA), matching the “slippery sequence” identified in ostrich (*Struthio camelus*) and turtles [54]. Specifically, the sequence forms a stem-loop structure between nucleotides 172–178 (5′-TTCCTAG-3′), facilitating ribosomal stalling and subsequent frameshifting. This mechanism skips the fourth nucleotide (position 174), merging codons 58 (TTC → Phe) and 59 (CTA → Leu) into a hybrid codon (TCT → Ser) to produce a contiguous NAD3 protein. Validated by homology-based Hidden Markov models(HMMs) [51], this finding supports the hypothesis that translational reprogramming, rather than genomic correction, is a conserved strategy in mitochondrial genome evolution.

Among the 22 tRNAs, tRNA^SER2(GCT)^ lacked the dihydrouridine hairpin structure, whereas all the remaining 21 tRNAs exhibited a canonical cloverleaf structure. The absence of a canonical cloverleaf structure in tRNA^SER^ has been observed in several animal species [36,55,56]. Several studies have shown that the absence of either the dihydrouridine arm or the thymidine–pseudouridine–cytidine (TΨC) loop in tRNASER may not impair its normal function [57,58]. These findings suggest that tRNA^SER2(GCT)^ is likely capable of performing its normal functions in *A. veredus*.

Our phylogenetic analyses of 88 Charadriiform species using ML and NJ methods recovered identical tree topologies, supporting the established division of Charadriiformes [11,12]. This study corroborates the division of Charadriidae into four monophyletic branches: *Pluvialis*, *Vanellus*, *Charadrius*, and *Anarhynchus*. This aligns with the Charadriidae classification framework proposed by Černý, D. and Natale, R. [11], who transferred the CRD II branch of the original *Charadrius* genus to the *Anarhynchus* genus. However, our study significantly improved branch support through a more complete mitochondrial dataset. Phylogenetic relationships among individual Charadriidae species are largely congruent with those of Sun et al. [12], though their study did not update the nomenclature for several *Anarhynchus* species previously categorized under *Charadrius*. Notably, our study supports the monophyly of *Anarhynchus*, which includes Oriental Plover (*A. veredus*), Mongolian Plover (*A. mongolus*), and *A. leschenaultii*, consistent with the classification revision of the International Ornithological Committee (IOC) based on naming priority. This study is the first to incorporate *A. veredus* into molecular phylogenetic analysis, unequivocally establishing its classification within *Anarhynchus* and supporting the IOC’s taxonomic revision. Additionally, the reclassification of the Lesser Sand Plover (*C. mongolus*) into two species—the Siberian Sand Plover (*C. mongolus*) and Tibetan Sand Plover (*C. atrifrons*)—is noteworthy. Finally, the basal position of *Pluvialis* within Charadriidae aligns with molecular systematic views that recognize *Pluvialis* as the independent subfamily Pluvialinae, further supporting its deep divergence (>20 Ma).

## 5. Conclusions

The *A. veredus* mitogenome exhibits conserved features relative to other Charadriidae species. Phylogenetic analyses confirm the well-established classification of Charadriiformes and support the monophyly of *Anarhynchus*, consistent with the IOC’s taxonomic revision. The identification of a conserved frameshift mechanism in *NAD3* underscores the role of translational reprogramming in mitochondrial genome evolution. These findings clarify the systematic position of *A. veredus* and provide novel insights into the diversification patterns within Charadriidae.

## Figures and Tables

**Table 1:** Characteristics of the mitochondrial genome of *A. veredus*.

Gene	Nucleotide Positions	Sizes (bp)	Strand	Intergenic Nucleotides	Start	Stop
*tRNA^PHE^*	1–71	71	+	-	-	-
*12s rRNA*	71–1049	979	+	−1	-	-
*tRNA^VAL^*	1050–1121	72	+	0	-	-
*16s rRNA*	1125–2725	1601	+	3	-	-
*tRNA^LEU^*	2718–2791	74	+	−8	-	-
*ND1*	2794–3771	978	+	2	ATG	AGG
*tRNA^ILE^*	3772–3841	70	+	0	-	-
*tRNA^GLN^*	3851–3921	71	−	9	-	-
*tRNA^MET^*	3921–3989	69	+	−1	-	-
*ND2*	3990–5030	1041	+	0	ATG	TAG
*tRNA^TRP^*	5031–5098	68	+	0	-	-
*tRNA^ALA^*	5100–5168	69	−	1	-	-
*tRNA^ASN^*	5170–5242	73	−	1	-	-
*tRNA^CYS^*	5246–5312	67	−	3	-	-
*tRNA^TYR^*	5312–5383	72	−	−1	-	-
*COX1*	5385–6935	1551	+	1	GTG	AGG
*tRNA^SER^*	6927–7000	74	−	−9	-	-
*tRNA^ASP^*	7006–7074	69	+	5	-	-
*COX2*	7076–7759	684	+	1	ATG	TAA
*tRNA^LYS^*	7761–7830	70	+	1	-	-
*ATP8*	7832–7999	168	+	1	ATG	TAA
*ATP6*	7990–8673	684	+	−10	ATG	TAA
*COX3*	8673–9456	784	+	−1	ATG	T
*tRNA^GLY^*	9457–9525	69	+	0	-	-
*ND3-CDS1*	9526–9699	174	+	0	ATC	-
*ND3-CDS2*	9701–9877	177	+	1	-	TAA
*tRNA^ARG^*	9880–9948	69	+	2	-	-
*ND4L*	9950–10,246	297	+	1	ATG	TAA
*ND4*	10,240–11,617	1378	+	−7	ATG	T
*tRNA^HIS^*	11,618–11,687	70	+	0	-	-
*tRNA^SER^*	11,688–11,753	66	+	0	-	-
*tRNA^LEU^*	11,753–11,823	71	+	−1	-	-
*ND5*	11,824–13,638	1815	+	0	GTG	TAA
*CYTB*	13,651–14,793	1143	+	12	ATG	TAA
*tRNA^THR^*	14,798–14,867	70	+	4	-	-
*tRNA^PRO^*	14,877–14,946	70	−	9	-	-
*ND6*	14,969–15,490	522	−	22	ATG	TAG
*tRNA^GLU^*	15,494–15,565	72	−	3	-	-
*D-loop*	15,566–16,886					

**Table 2:** Nucleotide composition and AT/GC skew of the *A. veredus* mitochondrial genome.

Region	Length (bp)	A%	T%	G%	C%	AT%	GC%	AT Skew	GC Skew
Genome	16,886	31.51	24.19	13.53	30.77	55.7	44.3	0.131	−0.389
PCGs	11,396	29.43	25.85	13.29	31.43	55.28	44.72	0.065	−0.406
tRNA	1546	30.08	28.01	20.83	21.09	58.09	41.91	0.036	−0.006
rRNA	2580	32.98	20.89	19.11	27.02	53.88	46.12	0.224	−0.171
D-loop	1321	31.79	28.69	12.41	27.10	60.48	39.52	0.051	−0.372

**Table 3:** Nucleotide composition of mitochondrial genomes in Charadriidae. Bold *Anarhynchus veredus* represented the complete mitogenome sequenced in this study.

Genus	Species	Length (bp)				GC Content (%)				AT Skew				GC Skew			
		Mitogenome	PCGs	rRNA	tRNA	Mitogenome	PCGs	rRNA	tRNA	Mitogenome	PCGs	rRNA	tRNA	Mitogenome	PCGs	rRNA	tRNA
*Pluvialis*	*P. apricaria*	15,723	11,388	2536	1551	45.4	45.9	46.1	41.7	0.15	0.081	0.22	0.051	−0.402	−0.424	−0.157	−0.002
	*P. fulva*	16,854	10,245	2562	1550	45.2	46	46.5	41.4	0.147	0.079	0.227	0.053	−0.395	−0.413	−0.169	−0.009
	*P. squatarola*	16,860	11,385	2560	1549	45.8	46.8	46.7	41.6	0.14	0.081	0.236	0.055	−0.382	−0.415	−0.17	−0.006
*Vanellus*	*V. cinereus*	17,074	11,394	2578	1551	44.9	45.3	45.6	42.6	0.147	0.082	0.231	0.047	−0.386	−0.418	−0.194	−0.005
	*V. vanellus*	16,795	11,391	2581	1551	44.6	44.8	45.8	42.7	0.134	0.078	0.234	0.042	−0.382	−0.41	−0.179	−0.006
*Anarhynchus*	*A. alexandrinus*	16,905	11,391	2578	1551	44.8	45.4	45.8	42.5	0.14	0.075	0.222	0.037	−0.389	−0.409	−0.162	−0.006
	*A. atrifrons*	16,919	11,385	2580	1551	44.5	44.9	45.9	42	0.153	0.06	0.222	0.031	−0.374	−0.403	−0.164	−0.005
	*A. leschenaultii*	16,905	11,388	2583	1553	44.5	44.6	46.3	42.1	0.135	0.066	0.235	0.044	−0.388	−0.403	−0.18	−0.008
	*A. mongolus*	16,844	11,388	2582	1547	44.7	44.6	46.2	42.2	0.131	0.067	0.235	0.043	−0.388	−0.404	−0.178	−0.009
	*A. montanus*	16,940	11,391	2580	1543	44.8	45.5	45.7	41.8	0.137	0.072	0.216	0.029	−0.381	−0.399	−0.155	−0.008
	***A. veredus***	16,886	11,394	2580	1546	44.3	44.7	46.1	41.9	0.131	0.065	0.224	0.036	−0.389	−0.406	−0.171	−0.006
*Charadrius*	*C. dubius*	15,933	11,397	2565	1552	45	45.3	45.7	41.5	0.139	0.08	0.232	0.032	−0.395	−0.406	−0.155	0.017
	*C. vociferus*	16,808	11,391	2576	1550	44.5	45.1	45.2	41.2	0.143	0.081	0.237	0.041	−0.398	−0.418	−0.168	0.005

## Data Availability

The datasets generated for this study are available in the Sequence Read Archive (SRA) of the NCBI database (Accession number: SRR32628449).

## Supplementary Materials

The following supporting information can be downloaded at: https://www.mdpi.com/article/10.3390/genes16091030/s1, Table S1: Taxonomic information and Genbank accession numbers for taxa used in the phylogenetic analysis; Table S2: Statistics of next-generation sequencing data.
